# Crystallization and preliminary crystallographic analysis of the major capsid proteins VP16 and VP17 of bacteriophage P23-77

**DOI:** 10.1107/S1744309112010330

**Published:** 2012-04-21

**Authors:** Ilona Rissanen, Alice Pawlowski, Karl Harlos, Jonathan M. Grimes, David I. Stuart, Jaana K. H. Bamford

**Affiliations:** a Department of Biological and Environmental Science and Nanoscience Center, University of Jyväskylä, PO Box 35, 40014 University of Jyväskylä, Finland; b Division of Structural Biology, Wellcome Trust Centre for Human Genetics, University of Oxford, Oxford OX3 7BN, England; c Science Division, Diamond Light Source Ltd, Diamond House, Harwell Science and Innovation Campus, Didcot OX11 0DE, England

**Keywords:** bacteriophages, capsid proteins

## Abstract

Members of the diverse double-β-barrel lineage of viruses are identified by the conserved structure of their major coat protein. New members of this lineage have been discovered based on structural analysis and we are interested in identifying relatives that utilize unusual versions of the double-β-barrel fold. One candidate for such studies is P23-77, an icosahedral dsDNA bacteriophage that infects the extremophile *Thermus thermophilus*. P23-77 has two major coat proteins, namely VP16 and VP17, of a size consistent with a single-β-barrel core fold. These previously unstudied proteins have now been successfully expressed as recombinant proteins, purified and crystallized using hanging-drop and sitting-drop vapour-diffusion methods. Crystals of coat proteins VP16 and VP17 have been obtained as well as of a putative complex. In addition, virus-derived material has been crystallized. Diffraction data have been collected to beyond 3 Å resolution for five crystal types and structure determinations are in progress.

## Abstract 2

The major capsid proteins VP16 and VP17 of bacteriophage P23-77 have been crystallized using both recombinant and purified virus and preliminary diffraction analyses have been performed.

## 1. Introduction

Analysis of virus structures determined over the past 30 years has led to the concept of ‘viral self’ elements that can be used to trace ancient evolutionary relationships (Benson *et al.*, 1999 ▶; Bamford *et al.*, 2005 ▶). ‘Viral self’ elements, vertically inherited elements that are required to assemble a virion, such as capsid proteins, are key to the survival of the virus; thus, while viral DNA may change, the structure and architecture of the virus and its capsid proteins are preserved. One such lineage, identified by conserved protein structures, containing adenovirus and the bacterial virus PRD1 traces its roots back to the divergence of the domains of life (Krupovic & Bamford, 2008 ▶; Bamford *et al.*, 2005 ▶). Modern members of this lineage are identified primarily by a capsid protein fold containing a double β-barrel. This structural signature has been observed in viruses such as adenovirus, PRD1, STIV, PBCV-1, PM2 and recently vaccinia virus (Abrescia *et al.*, 2004 ▶, 2008 ▶; Bahar *et al.*, 2011 ▶; Khayat *et al.*, 2005 ▶; Nandhagopal *et al.*, 2002 ▶; Rux *et al.*, 2003 ▶; Liu *et al.*, 2010 ▶; Reddy *et al.*, 2010 ▶), that infect hosts from different domains of life.

P23-77 is a bacteriophage which appears to possess structural elements that bear a novel relationship to the adeno/PRD1 lineage. It has a *T* = 28 icosahedral capsid, ∼800 Å across, with an internal lipid membrane enclosing a circular dsDNA genome (Yu *et al.*, 2006 ▶; Jaatinen *et al.*, 2008 ▶; Jalasvuori *et al.*, 2009 ▶). The genome is approximately 17 000 nucleotides in length and contains 37 putative ORFs, ten of which code for structural proteins (Jaatinen *et al.*, 2008 ▶; Jalasvuori *et al.*, 2009 ▶). P23-77 has two major coat proteins, VP16 (20 kDa) and VP17 (32 kDa); they form, in an approximate 1:1 ratio, a viral capsid with an unusual base-and-tower architecture reminiscent of the capsomeric structure observed in some members of the adeno/PRD1 lineage but sited at positions in the icosahedral virus that are not allowed for the adeno/PRD1 trimeric capsomers. Thus, while in the adeno/PRD1 lineage the pseudo-hexameric capsomers are formed by trimers composed of subunits harbouring a double-β-­barrel fold, in P23-77 some capsomers lie on icosahedral twofold axes. This organization has only recently been identified in extremo­phile viruses and has not been characterized at the level of protein structure (Jaatinen *et al.*, 2008 ▶; Jäälinoja *et al.*, 2008 ▶). In addition, sequence analysis of the packaging ATPase of P23-77 suggests a relationship to the adeno/PRD1 double-β-barrel lineage. P23-77 may belong to an unknown ancestral branch of the double-β-barrel lineage that utilizes single-β-barrel core structures instead of the duplicate version and has modern members among extremophile viruses, including the archaeal virus SH1 (Jalasvuori *et al.*, 2009 ▶, 2010 ▶). Here, we report the crystallization and preliminary diffraction analysis of VP16 and VP17, with the aim of ultimately solving their structures by X-ray crystallography, in order to shed light on the origins of P23-­77 and its relatives.

## 2. Materials and methods

### 2.1. Plasmid construction

Genes *ORF16* and *ORF17* were PCR-amplified from the P23-77 genome using primers with restriction sites for *Nde*I and *Hin*dIII and corresponding to full-length VP16 (1–173) and VP17 (1–291), respectively. Purified restricted PCR fragments were ligated with *Nde*I–*Hin*dIII-restricted expression vector pET22b(+) (Novagen) and the resulting recombinant plasmids pIR1 (ORF17/pET22b) and pIR2 (ORF16/pET22b) were used to transform competent *Escherichia coli* HMS174 (DE3) for high-level recombinant protein expression using standard methods.

### 2.2. Protein expression and purification

Large-scale cultures of *E. coli* HMS174 (DE3) transformed with each plasmid were grown (12 × 400 ml LB medium with 150 µg ml^−1^ ampicillin, 310 K, 230 rev min^−1^) until the absorbance at 550 nm reached 0.5, at which point recombinant protein expression was induced by the addition of IPTG to a final concentration of 1 m*M*. Cultures were grown for 22 h. Cells were collected by centrifugation, resuspended to one hundreth of the original volume in 20 m*M* Tris pH 7.4, 50 m*M* NaCl buffer and disrupted with a French press (Thermo Fisher Scientific). Soluble fractions containing target proteins were seperated from cell debris by centrifugation (Beckman Ti-70 rotor, 33 000 rev min^−1^, 2 h, 278 K).

Supernatants containing VP16 or VP17 were incubated at 363 K for 10 min, which caused degradation of the less heat-stable host-derived proteins. Degraded material was removed by centrifugation, after which samples were concentrated and buffer-exchanged (20 m*M* ethanolamine pH 9, 50 m*M* NaCl for VP16 and 20 m*M* ethanolamine pH 9.5 for VP17) using an Amicon ultrafiltration system (Millipore).

VP16 was loaded onto an anion-exchange chromatography column [5 ml Q HP HiTrap column (GE Healthcare) equilibrated with 20 m*M* ethanolamine pH 9 at 295 K]. The flowthrough containing VP16 was further purified by size-exclusion chromatography [HiLoad 26/60 Superdex 200 prep-grade column (GE Healthcare) equilibrated with 20 m*M* Tris pH 7.4, 150 m*M* NaCl at 295 K]. VP17 was purified with a similar anion-exchange chromatography protocol in which the column was equilibrated with 20 m*M* ethanolamine pH 9.5 and the protein was eluted specifically with 50 m*M* NaCl. Fractions containing VP17 were purified by size-exclusion chromatography as for VP16. After size-exclusion chromatography, fractions containing pure VP16 or VP17 were pooled, concentrated, exchanged into 20 m*M* Tris pH 7.4 buffer and stored at 280 K. Purified VP16 and VP17 were concentrated using 10 kDa molecular-weight microconcentrators (Amicon).

### 2.3. P23-77 virus purification

Virus particles were produced in *T. thermophilus* strain ATCC33923 and purified as described previously (Jaatinen *et al.*, 2008 ▶). In brief, cells were infected at a cell density of 7 × 10^8^ cfu ml^−1^ with a multiplicity of infection (MOI) of around 10. Viral particles were precipitated from the lysate with 12% polyethylene glycol (PEG) 6000 and 0.5 *M* NaCl and concentrated to one twentieth of the original lysate volume in TV buffer (20 m*M* Tris–HCl pH 7.5, 5 m*M* MgCl_2_, 150 m*M* NaCl). Viruses were purified by rate zonal centrifugation [linear 5–20%(*w*/*v*) sucrose gradient in TV buffer, 23 000 rev min^−1^, 45 min, 298 K], followed by equilibrium centrifugation in 1.30 mg ml^−1^ CsCl_2_ in TV buffer (21 000 rev min^−1^, 16 h, 298 K). 2× purified viral particles were collected by differential centrifugation (32 000 rev min^−1^, 4 h, 298 K) and the virus pellet was suspended in TV buffer. Purified virus samples were stored at 295 K.

## 3. Results

### 3.1. Crystallization

Crystallization conditions for purified full-length VP16 (1–173) and full-length VP17 (1–291) and the P23-77 virion were initially screened by hanging-drop vapour diffusion using a Mosquito Nanodrop Crystallization Robot (TTP LabTech) at the University of Jyväskylä, Finland. Subsequently, final crystallization experiments were performed at the Division of Structural Biology, Oxford University, England either by setting up crystallization experiments manually or by using the nanolitre high-throughput facility with sitting-drop sizes of 1 µl + 1 µl and 100 nl + 100 nl (protein solution and crystallization screen), respectively (Walter *et al.*, 2005 ▶). Commercially available crystallization screening kits (Hampton Research, California, USA, Molecular Dimensions, UK and Emerald BioStructures, Washington, USA) were used for all initial experiments. 576 crystallization conditions were tested for VP16 and 288 conditions were tested for VP17, whilst the VP16–VP17 complex was screened against 984 conditions. Virion crystallization was tested against 480 conditions. All crystallizations were set up at room temperature (293–295 K). A number of different crystal forms were obtained.

VP16 type 1 crystals grew within 1–2 weeks from microlitre drops of protein (2–3 mg ml^−1^) mixed in a 1:1(*v*:*v*) ratio with a solution consisting of 5%(*w*/*v*) PEG 1000 and 5%(*w*/*v*) PEG 8000 dissolved in autoclaved water (the crystallization drop was pH 7.4). Additional screening experiments carried out in 96-well plates in the high-throughput crystallization facility (Walter *et al.*, 2005 ▶) yielded VP16 type 2 crystals. These crystals grew within days from 20%(*w*/*v*) polyethylene glycol 6000, 0.1 *M* citrate pH 4.

VP17 crystals were obtained from drops consisting of 1 µl protein solution (2–3 mg ml^−1^) mixed with 1 µl 1.9 *M* sodium formate, 0.1 *M* bis-Tris buffer pH 7.0. Crystals grew to full size in two weeks.

In an attempt to obtain the structure of a VP16–VP17 complex, VP16 and VP17 were mixed at concentrations of 1.7 and 2.0 mg ml^−1^, respectively (*i.e.* a 1:1 molar ratio); one well diffracting crystal, which was used for data collection, took three months to grow from 1.1 *M* diammonium tartrate pH 7.

**Figure 1:**
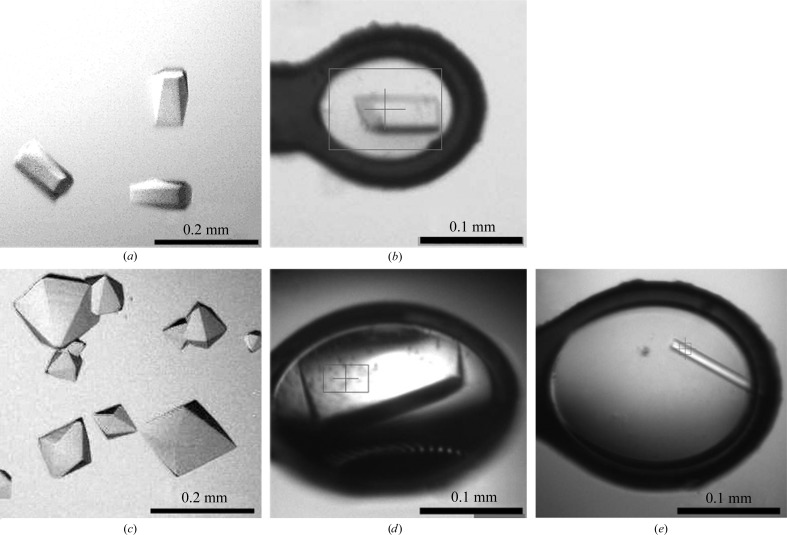
Crystals of the major coat proteins of P23-77: (*a*) VP16 type 1, (*b*) VP16 type 2, (*c*) VP17, (*d*) putative complex, (*e*) virus-derived crystals. (*b*), (*d*) and (*e*) show crystals frozen in loops that were used for data collection at the beamline.

Crystals were also obtained from experiments using the whole P23-­77 virion. Crystallization conditions were screened in 96-well plates with virion concentrations ranging from 1.0 to 2.5 mg ml^−1^. Thin needles appeared in various conditions, all of which contained 0.1 *M* citric acid pH 3.5 and PEG. Crystals appeared within 12 h to 8 d depending on the buffer conditions, virion concentration and virus preparation. Crystals used for diffraction analysis were grown in microlitre drops which were set up manually at a virus concentration of 2.4 mg ml^−1^ and a virus:reservoir ratio of 1:1(*v*:*v*). The reservoir solution consisted of 0.1 *M* citric acid pH 3.5, 20 m*M* Tris–HCl pH 7.5, 5 m*M* MgCl_2_, 150 m*M* NaCl and 25% PEG 3350. All five crystal types used for diffraction analysis of VP16, VP17, their complex and virion-derived material are illustrated in Fig. 1 ▶.

### 3.2. X-ray characterization and data collection

All crystals were cooled in liquid nitrogen using glycerol mixed with reservoir solution at 25%(*v*/*v*) as a cryoprotectant and exposed to X-rays at 100 K. Initially, crystals of VP16 type 1 and VP17 were characterized at Jyväskylä and Oxford using in-house X-ray equipment. Subsequently, definitive data sets for all crystals were collected at the Diamond Light Source synchrotron, Didcot, England as follows. VP16 type 1 data were collected on beamline I03 (λ = 0.979 Å) in high- and low-resolution sweeps, with the high-resolution sweep consisting of 360 images with 0.5° oscillation per image and an exposure time of 1 s per image. VP16 type 2 data were collected on beamline I04 (λ = 1.000 Å) as 360 images with 1° and 1 s per image. VP17 data were collected on beamline I04 (λ = 1.000 Å) as 614 images with 0.5° and 1 s per image. The data for the putative complex were collected on beamline I24 (λ = 1.071 Å) as 1800 images with 0.2° and 0.2 s per image. Data for the virion-derived crystal were collected on beamline I24 (λ = 0.969 Å) as 2 × 450 images with 0.2° and 0.2 s per image. All crystals diffracted to better than 3 Å resolution. Data were automatically processed with* xia*2/*XDS* (Winter, 2010 ▶; Kabsch, 1993 ▶) and the processing statistics are summarized in Table 1 ▶.

## 4. Discussion

P23-77 major capsid proteins VP16 and VP17 have been purified, crystallized and native data sets collected. The host of bacteriophage P23-77 is the extremophile *T. thermophilus* and consequently the thermal stability of the P23-77 proteins made purification straightforward; raising the temperature of the clarified supernatant to 363 K for 10 min degraded and precipitated most cellular proteins.

Preliminary data analyses indicate that the asymmetric units of VP16 type 1 and VP16 type 2 are likely to contain one subunit of VP16 each (corresponding to solvent contents of 65 and 41%, respectively); the asymmetric unit of VP17 probably contains two subunits of VP17 (corresponding to a solvent content of 59%) and the asymmetric unit of the putative complex could accommodate one subunit each of VP16 and VP17 (corresponding to a solvent content of 40%). The unit cell of the crystal derived from virus crystallization is far too small to contain the whole virus (which is some 800 Å across, exceeding a complete unit cell in every direction). It is very likely to contain VP16 and/or VP17 (for example, six subunits of VP16 in the crystallographic asymmetric unit would correspond to a solvent content of 55%, whereas four subunits of VP17 would correspond to 52% solvent). A search for heavy-atom derivatives is in progress. Structures of major capsid proteins VP16 and VP17, which are the building blocks of P23-77, and their complexes will provide details of both their oligomeric states and how they assemble to form part of the capsid of P23-77. This will contribute to the overall picture of the evolutionary relationships in this diverse group of dsDNA viruses.

## Figures and Tables

**Table 1:** Data-collection and processing statistics Values in parentheses are for the highest resolution shell. Each data set was collected from one crystal, except for the virus-derived crystal data set, which was collected from two.

	VP16 type 1	VP16 type 2	VP17	Putative complex	Virus-derived crystals
Space group	*P*6_2_22	*C*2	*P*6_1_22	*C*2	*P*2_1_2_1_2_1_
Unit-cell parameters					
*a* (Å)	61.9	76.6	107.2	76.8	41.8
*b* (Å)	61.9	68.6	107.2	69.6	78.2
*c* (Å)	251.2	31.6	233.8	81.6	405.8
α (°)	90	90	90	90	90
β (°)	90	96.4	90	105.0	90
γ (°)	120	90	120	90	90
Resolution (Å)	62.8–1.80 (1.85–1.80)	34.3–1.26 (1.30–1.26)	59.7–2.26 (2.32–2.26)	39.6–1.53 (1.57–1.53)	51.2–2.92 (2.99–2.92)
*R*_merge_†	0.075 (1.045)	0.053 (0.626)	0.082 (0.917)	0.064 (1.015)	0.301 (1.492)
〈*I*/σ(*I*)〉	30.7 (3.3)	23.1 (2.6)	38.6 (5.3)	17.7 (2.9)	6.4 (1.5)
Completeness (%)	100 (100)	85.8 (41.4)	100 (100)	98.4 (83.3)	99.9 (99.8)
Multiplicity	28.5 (21.0)	7.5 (6.5)	35.5 (36.6)	6.3 (4.4)	6.3 (5.6)

† *R*_merge_ = 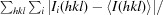 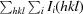, where *I*_*i*_(*hkl*) is the *i*th measurement and 〈*I*(*hkl*)〉 is the weighted mean of all measurements *I*_*i*_(*hkl*).
